# Basalt Fiber Reinforced Concrete: A Compressive Review on Durability Aspects

**DOI:** 10.3390/ma16010429

**Published:** 2023-01-02

**Authors:** Buthainah Nawaf Al-Kharabsheh, Mohamed Moafak Arbili, Ali Majdi, Saleh M. Alogla, Ahmad Hakamy, Jawad Ahmad, Ahmed Farouk Deifalla

**Affiliations:** 1 Civil Engineering Department, Faculty of Engineering, Al-Albayt University, Mafraq 25113, Jordan; 2 Department of Technical Civil Engineering, Erbil Technical Engineering College, Erbil Polytechnic University, Erbil 44001, Iraq; 3 Department of Building and Construction Techniques, Al-Mustaqbal University College, Hillah 51001, Iraq; 4 Department of Civil Engineering, College of Engineering, Qassim University, Buraydah 51452, Saudi Arabia; 5 Department of Physics, Faculty of Applied Science, Umm Al-Qura University, Makkah 21955, Saudi Arabia; 6 Department of Civil Engineering, Military College of Engineering, Sub Campus of National University of Sciences and Technology, Islamabad 44000, Pakistan; 7 Structural Engineering Department, Faculty of Engineering and Technology, Future University in Egypt, New Cairo 11845, Egypt

**Keywords:** basalt fibers, shrinkage, thermal properties, scan electronic microscopy

## Abstract

The creation of sustainable composites reinforced with natural fibers has recently drawn the interest of both industrial and academics. Basalt fiber (BF) stands out as the most intriguing among the natural fibers that may be utilized as reinforcement due to their characteristics. Numerous academics have conducted many tests on the strength, durability, temperature, and microstructure characteristics of concrete reinforced with BF and have found promising results. However, because the information is dispersed, readers find it problematic to assess the advantages of BF reinforced concrete, which limits its applications. Therefore, a condensed study that provides the reader with an easy route and summarizes all pertinent information is needed. The purpose of this paper (Part II) is to undertake a compressive assessment of basalt fiber reinforced concrete’s durability features. The results show that adding BF significantly increased concrete durability. The review also identifies a research deficiency that must be addressed before BF is used in practice.

## 1. Introduction

In recent years, there have been increased demands for strength and durability features due to the construction of large-scale infrastructure in several challenging service settings [1,2,3,4]. Contrarily, concrete has several unfavorable characteristics, including brittleness, low impact resistance, and excessive weight. Consequently, there is a need to improve tensile capacity [5,6,7,8]. One of the current hot topics for building materials research is the use of fiber-reinforced technology to boost the durability of concrete [9,10,11]. Traditional concrete is typically strong in compression, but not in tension [12,13,14]. Reinforcement bars are frequently utilized in concrete to compensate for the tensile stresses. In fiber-reinforced concrete, a specific type of concrete, fibers are added to increase the necessary tensile capacity of concrete [15,16].

Since ancient times, many parts of the world have been using fibers in construction materials. The motivation for this effort was to boost the tensile strain of the concrete’s “perceived” delicate properties. In the 20th century, this technique was employed to produce fiber-reinforced concrete, which has grown in popularity and apply in construction sector due to its improved strength. The components of concrete are reinforced with a variety of fibers, including biological and inorganic fibers. The surface of the fibers, length, elastic modulus, and the material from which they are made all perform a role in determining the type of fibers used in concrete to increase tensile strength. Additionally, it is uncertain to what degree these fibers affect the functionality of concrete [17]. Metallic and nonmetallic fibers are the two categories into which fibers are frequently categorized. The capacity of metallic fibers to conduct electricity sets them apart from nonmetallic fibers. Steel fibers make up the majority of metallic fibers, whilst nonmetallic fibers are made of materials like steel fiber [18], propylene [19], carbon [20], jute fiber [21], glass fibers [22] and other similar ones [23]. Concrete brittleness may be reduced by adding fiber, and its strength and durability can be increased [13,24,25,26,27].

**Figure 1:**
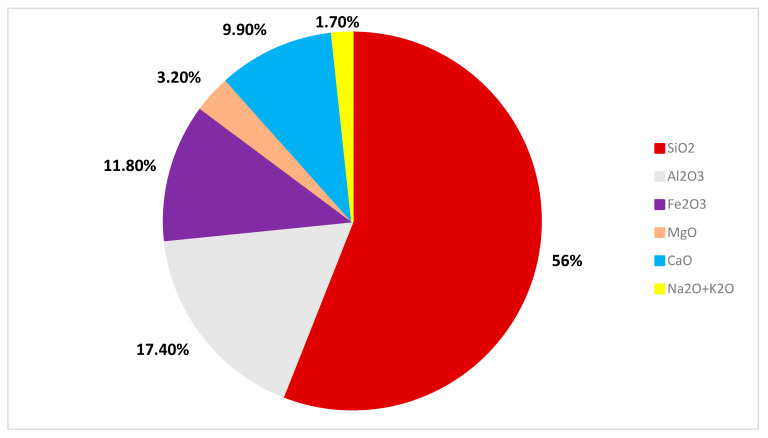
Chemical composition of BF [29].

BFs are made from genuine basalt ore using hot melting and wire drawing techniques at high temperatures [28]. When the chemical composition is examined, it can be shown that SiO_2_ is the primary component and Al_2_O_3_ is the second, as indicated in Figure 1.

**Figure 2:**
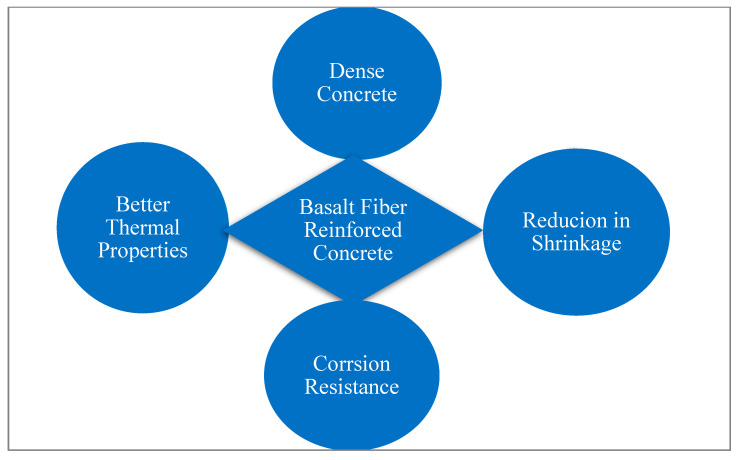
Benefits of BF.

The kind, quality, and production method of the raw materials, as well as the qualities of the finished product, all influence the base cost of BFs. The chemical and mechanical qualities are influenced by the composition of the raw materials, much like the price. Variations in composition and element concentration result in variations in thermal and chemical stability as well as favorable strength and basic qualities. Generally, the production of this type of fiber is comparable to that of glass fiber (GF), but it uses less energy and does not include any additives, making it less expensive than GF or carbon fiber (CF). Basalt rock from volcanic eruptions is used as the raw material to make BF, which is then molten in a kiln at 1450–1500 °C. The heated substance is then driven through a platinum/rhodium crucible bushing to produce fibers. Constant spinning is a technique that may provide reinforcing material in the form of continuous fibers or chopped fibers for use in the manufacture of textiles and has a lot of potential users in composite materials. Along with being simple to handle using standard procedures, it also has significant cost advantages [30]. The advantages of BF used in concrete are shown in Figure 2.

A new class of inorganic fiber called BF has several benefits, including strength, stability, insulation, corrosion resistance, simple production, cheap cost, and high compatibility [31]. The insulating capabilities and anti-corrosion of BF make it further appropriate for extreme-speed rail and road engineering than those of metal fiber. BF is an eco-friendly material as well [32].

Every year, significant economic losses and security risks are brought on by steel corrosion in concrete buildings. In salty conditions, chloride damage is a virulent reason for steel corrosion [33]. According to research [34], BF is more resistant to corrosion in saltwater than carbon and GF. Additionally, some researchers noted that the BF’s alkali resistance was low, in contrast to these investigations. According to Lee’s research [35], the weight loss rate in strong alkaline solutions may reach 40%. The strength loss rate of BF is as great as 80% after 90 days of immersing in an alkaline mixture. According to research, BF concrete performed better than CF and GF concrete. Engineering mechanics and durability may be enhanced by BF [30].

According to the findings, self-compacting concrete is less practical when BFs are added. The physical qualities of concrete might be greatly improved, but BF decreased the flow properties [36]. The impacts of polypropylene and BFs on the mechanical parameters of concrete samples were examined. The tensile strength (TS) and flexural strength (FS) of specimens of concrete were claimed to be significantly increased by fiber. It is not immediately clear, nevertheless, how compressive strength (CS) improves [37]. Excellent mechanical and physical properties of BFs include good resistance to corrosion, excellent heat resistance, and resistance to alkalis and acids [30]. The three-dimensional network that was created by the irregular BF mixing in the concrete is closely connected to the cement paste and aggregate. A small amount of porosity reduction, a delay in the appearance of tiny internal fissures, and a more compact structure are all possible effects of doped fiber [10]. According to research, reinforced concrete with fiber reinforcement (BFRC) has a greater bonding capacity with steel bars in saline-alkaline environments and a greater level of dependability in bonding slip tests [38]. According to a study on the chemical stability of BF in alkaline mixtures, BF immersed in a mild alkaline mixture could be extremely durable, with a minimal weight-loss rate after immersing in a Ca(OH)_2_ mixture for three months [35].

Many scholars work on strength properties, durability aspects, thermal properties, and microstructure analyses of BFs-reinforced concrete and stated an encouraging response. However, information is dispersed, and readers cannot simply judge the benefits of BFs-reinforced concrete, which restricts its uses. Therefore, a compressive study is required that summarizes all relevant information and provides an easy path for the reader. Although a compressive review was conducted by other scholars [39], some durability properties are missing such as shrinkage, density, and electrical resistivity. The aim of this article is to conduct a compressive review on durability aspects such as density, water absorption, electric resistivity, freezing and thawing effects, chloride content, shrinkage, rapid chloride ions penetrability, and alkali resistance. The results indicate that the durability assets considerably enhanced with the addition of BF. Furthermore, the review also suggests recommendations that must be explored before being used practically.

## 2. Apparent Density

**Figure 3:**
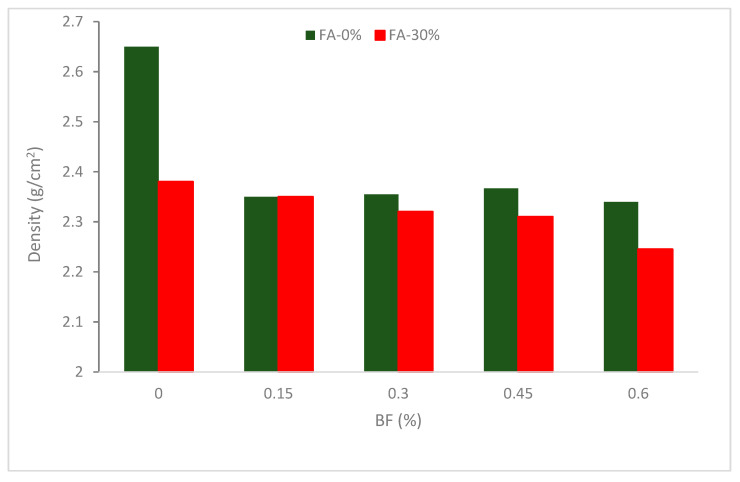
Density of basalt fiber (BF) reinforced concrete: Source [40].

Figure 3 displays each mixture’s apparent density. The findings demonstrate a declining tendency in perceived density with boosting BF percentages. The density of concrete reduces with increasing BF percentage even though BF has a slightly greater density than concrete. The following is a summary of the causes: First, BF may result in a weak matrix across the fiber and reduce compatibility. Second, the BF network configuration produced prevents cement paste from separation and movement, making it more difficult to remove voids and bubbles with shaking. Third, BF needs an additional plasticizer to sustain the target slump, and the plasticizer can cause more air cavities because it includes the elements of the air-entrain. All of these factors cause more air spaces to exist in concrete, and this fact plays a significant role in determining the material’s apparent density [25].

Additionally, BF creates a network structure that boosts the matrix’s internal binding force and prevents cement paste from flowing or segregating. Therefore, the presence of BF causes concrete’s flowability to decrease [41], making it difficult to fill up gaps in concrete using vibration. Due to this, the interior sponginess rises and the compatibility falls, which lowers the apparent density. Furthermore, with the same fiber content, the density of a combination containing secondary cementitious materials (SCM) is constantly smaller than it would be without them. This may be attributable to cement’s greater specific gravity than fly ashes. A lower fresh concrete density and porous concrete are the results of higher fiber dosages (4.0%), which makes the compaction activity additional challenging. Concrete gains density by around 15% when 1.5 percent of its volume is added to fibers [42]. Research revealed that the permeability coefficient of concrete is decreased by the addition of BF. The permeability coefficient reduces by 86.3, 85.5, and 84.3 percent when the volume content of BF is 0.1, 0.2, and 0.3 percent, respectively [43].

It can be concluded that the density of concrete decreased with the addition of BF. However, with the substitution of cementitious materials such as fly ash, a slightly decreased in density was observed up to 0.45% addition of BF, further addition of BF (0.6%) results in considerably decreased density.

## 3. Permeability

### 3.1. Sorptivity

Hazardous chemicals like chloride and sulphate ions may penetrate construction materials via water infiltration. Diffusion and capillary action are the two major methods of transporting chloride and sulphate ions through the material, although transformation by itself is a very sluggish procedure. Consequently, particularly close to the unsaturated concrete surface, capillary action could be the primary transport pathway. Understanding how concrete transports moisture is crucial for determining its service life and enhancing its value as a construction material [44].

**Figure 4:**
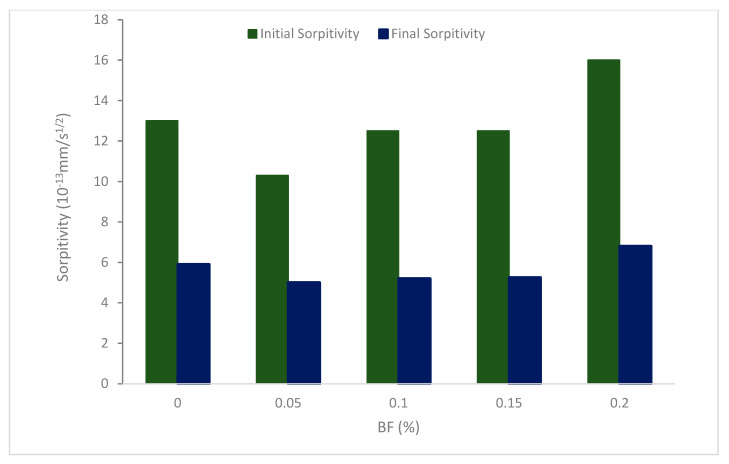
Sorpitivity of BF reinforced concrete: Source [46].

The total water absorption over 6 h and 8 days, respectively, was used to calculate the initial and secondary sorptivity. Figure 4 displays the sorptivity coefficients of concrete. A similar pattern can be seen in both the primary and secondary sorptivity of all concrete mixtures. BF5 (0.05%) has the lowest sorptivity, while BF20 (0.20%) has the maximum. The initial and secondary sorptivity of the specimen with 0.05 percent t o0.15 percent BF was lowered, falling by 2.75 to 20.97 percent, and 5.39 to 16.78 percent, respectively, associated with the control concrete. The initial and secondary sorptivity are boosted by 19.35 and 20.52 percent, respectively, by a BF concentration of 0.2 percent. It is determined that lowering water absorption and sorptivity depends significantly on the presence of suitable BF. When steel slag is replaced with zigzag-shaped fibers, the porosity of the concrete is reduced, which reduces capillary action in steel slag concrete and improves the resistance of the movement of water through it. Compared to traditional concrete, the elements in the concrete mix are tightly packed and have less porosity. The value of the coefficient of sorptivity is 10.67% smaller in fiber-reinforced concrete than in regular concrete, per the test findings [45]. It can be concluded that sorpitivty decreased with the addition of BF due to the crack prevention of BF. However, a higher dose of BF (0.2%) results in considerably increased sorpitivity value due to lack of flowability, which increased compaction efforts, leading to more voids.

### 3.2. Water Absorption (WA)

Concrete must have both compressive strength and long-term durability. The density of concrete has an impact on its long-lasting qualities. Compact (dense) concrete has more load carrying capacity and has fewer voids and pores. Concrete with fewer voids is less porous to liquid chemicals and water. As a result, this kind of concrete will last longer, and less WA or other harmful chemicals will penetrate.

**Figure 5:**
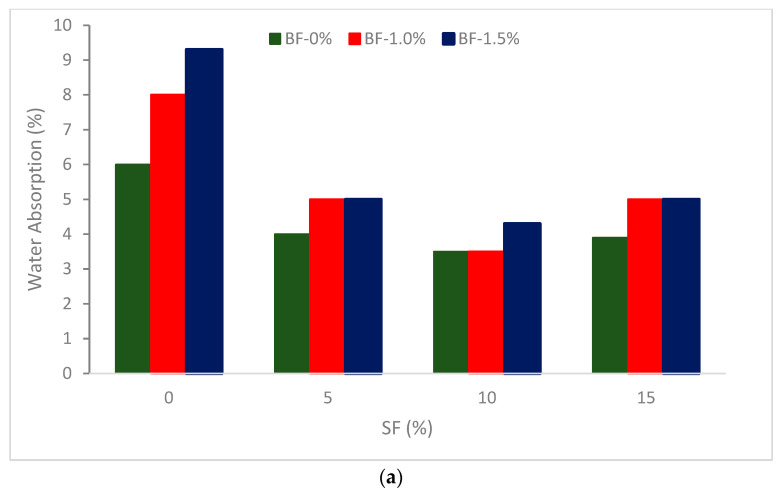

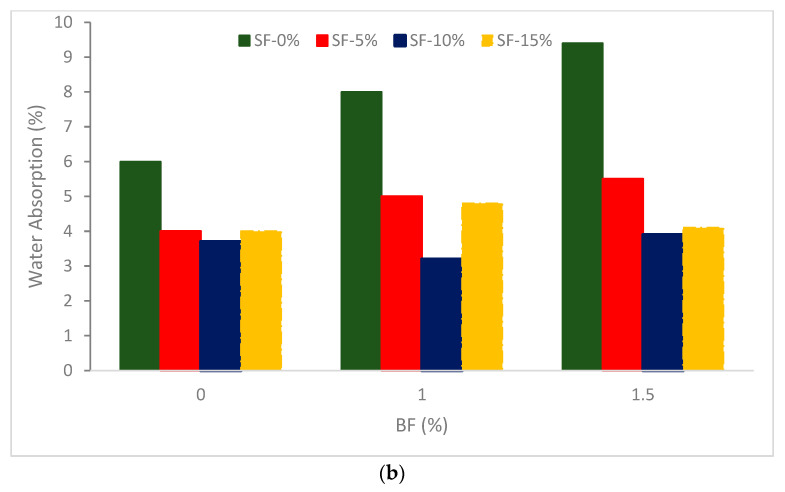
Water absorption of concrete (**a**) SF at BF 0,1 and 1.5%: (**b**) BF at SF 0,5,10 and 15%: Source [41].

A study [41] shows that the specimens with 10 percent silica fume have the lowest values of water absorption, which are 3.22 percent, 3.36 percent, and 4 percent for specimens with 0 percent, 1 percent, and 1.5 percent basalt, respectively. Figure 5 demonstrates a 44 percent, 43 percent, and 32 percent drop from the control specimen.

Glass fibers (GF) had a far better effect on water absorption (WA) than polypropylene fibers. Adding 1.35 percent GF decreased the sample WA from 3.49 percent to 1.99 percent, with a maximum reduction of 43.1 percent, when the water/binder ratio was 0.30. The highest decline of 10.6 percent in water absorption (WA) was achieved by incorporating 0.45 percent PPF, which decreased it from 3.49 to 3.12 percent [47].

The statistics on water absorption show that increasing the fiber content somewhat improves the WA as associated to reference SCC. Nevertheless, the water absorption values of all fiber-reinforced SCC were within 2% of one another. Concrete has less water penetration because of a more compact pore structure as a consequence of the GF’s tight bond with cement particles. The endurance of the concrete is increased by the introduction of GF [48]. It was known that the least amount of WA was attained at 2.0% steel fiber [49]. Regular concrete has a lower MOE than fiber concrete. Fibers would improve the tensile strain properties of concrete, reducing the development and spread of early fractures [50]. Therefore, BF addition decreased water absorption penetration due to crack prevention. Furthermore, the substitution of secondary cementitious materials such as silica fume considerably due to combined pozzolanic and micro filling effects. However, some higher dose results in increased water absorption due to a lack of flowability, which increased compaction efforts, leading to more possibility of voids which results in more water absorption. Therefore, the review recommends a higher dose of superplasticizer for a higher dose of BF addition.

### 3.3. Rapid Chloride Ions Penetrability

**Figure 6:**
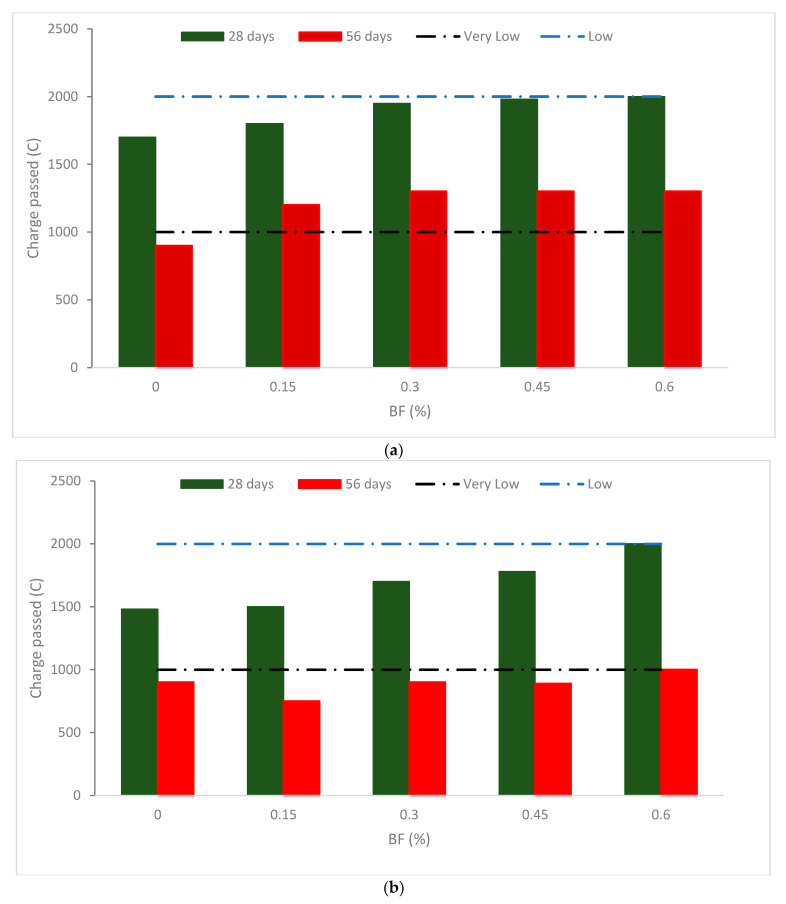
Charge pass of BF reinforced concrete (**a**) 0% fly ash and (**b**) 30% fly ash: Source [40].

Figure 6 displays the outcomes of the charge after it was run through all combinations that were aged between 28 and 56 days. The outcomes show that adding BF raises the charge of the concrete. For the same series, a greater charge is obtained in the blends with greater BF (0.60 percent). According to a study [41], the inclusion of BFs in cementitious mixtures leads to a reduction in electrical resistivity, making electrical resistivity more apparent as fiber volume increases. Results from the electrical resistivity test have been altered by the percentages and composition of pores and the chemical composition of the pore mixture. Therefore, specimens containing 15% silica fume do not necessarily have higher performance or durability because of their higher specific electrical resistivity. According to Zhang et al. [51], adding BF enhances the permeability of concrete because the fiber causes an increase in porosity. Other organic fibers, such as polypropylene fibers were explored in earlier research, which suggested a poorer chloride resistance with fibers in contrast with no fiber [52]. Though, according to a study [36], adding BF makes self-compacting concrete more resistant to chlorides and decreases porosity.

The defects generated by BF, such as fractures and pores surrounding fiber in the matrix, are responsible for the charge rise [53]. Particularly, the porous, weak fiber-matrix interfacial transition zone (fiber-ITZ). The fibers become more easily clumped and unevenly distributed as the BF content rises, and more severe flaws develop as a result of the tangled and folded fibers. Moreover, fibers have a significant surface area, and further cement paste is required to coat them [54].

The total charge transmitted through BF reinforced with 30% fly ash (FA) falls by 7.4 percent to BF without fly ash. Fly ash (FA) and silica fume (SF) both drop by 20.3 percent and 50.7 percent after 56 days. This finding suggests that the charge of BF-reinforced concrete is reduced when mineral admixtures are added, particularly when fly ash and silica are combined together. The pozzolanic activity and pore-filling properties of SCM are responsible for this alteration [6]. Mineral admixtures’ ability to bind chloride reduces the charge that passes through the concrete [55]. Additionally, SF has a significantly smaller grain size than FA, which intensifies the pozzolanic reaction and pore-filling effects.

According to Thomas et al. [56], ternary cementitious mixes provide concrete with a very high chlorine resistance. A similar pattern is seen as the curing age rises. For basalt fiber reinforced concrete, the total charge transmitted through mixes after 56 days reduces by 36.8% as compared to those at 28 days. Additionally, when the curing age increases, the charge with cementitious dramatically reduces. It may be related to the fact that the pozzolanic activity early phase is delayed and the result is not immediately apparent. Though, when concrete ages, an intense pozzolanic reaction produces a considerable quantity of secondary hydration products that greatly enhance concrete’s compactness. As a result, as people age, the positive effects of mineral admixtures become more noticeable. In contrast, the research found that adding 1, 1.5, or 2% BF to a concrete mixture in which 40% fly ash replaces some of the cement increases the combination’s resistance to the chloride penetration-associated reference [57]. It can be concluded that chloride resistance decreased considerably with the addition of BF due to the crack prevention of BF. The resistance further increased with the substitution of pozzolanic materials (fly ash), which fill the voids among concrete ingredients, leading to more dense concrete. Additionally, due to the pozzolanic reaction, secondary cementitious compounds such as calcium silicate hydrates (CSH) are formed, which increase the binding properties of cement paste.

## 4. Shrinkage

Shrinkage is seen in the early hours after casting, however, it may be prevented by improving the blend mix and proper curing. Carbon dioxide and solidified cement paste combine to induce carbonation shrinkage [58].

**Figure 7:**
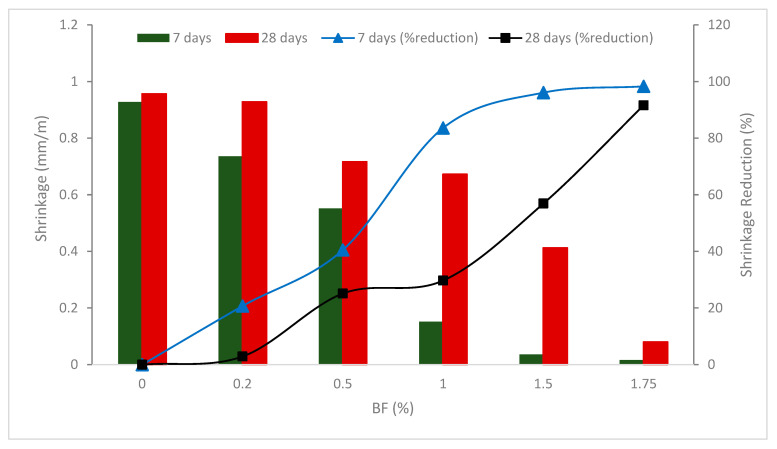
Shrinkage of BF reinforced concrete: Source [59].

According to the experimental findings, depending on the fiber content, using BFs greatly reduced concrete shrinkage and increased efficiency. Figure 7 depicts the shrinkage reduction impact of BF after 7 days of concrete curing. The findings demonstrated that concrete shrinkage dramatically reduced as the fiber concentration rose. The shrinkage reduction efficiency in concrete employing 0.5 percent fiber compared to the control achieved 40% in the first week. The shrinkage reduction impact of concrete is quite strong, reaching 84 percent in comparison to the control when employing fibers up to 1.0 percent. The effectiveness of concrete shrinkage reduction reached 98 percent when the fiber content was raised to 1.75 percent.

The concrete shrinks by 0.716 mm/m, which is 25% less than the control sample, when the fiber content is utilized at 0.5 percent. The shrinkage will be reduced by 56.9% and 91.6 percent, respectively, associated with the reference, with the fiber content raised to 1.5 and 1.75 percent. When fibers are added to concrete mixtures, shrinkage in concrete is improved. A reduction in the amount of moisture in the concrete and shrinkage of the concrete will occur along with the hydration of the cement. However, the fiber will play a role as a bridge to transport stress throughout the concrete in the presence of micro-sized BF, considerably decreasing shrinkage. A study also reported that carbon nanotubes delay the propagation of micro-cracks and strengthen the interfacial transition zone [60]. However, the shrinkage reduction impact for concrete does not significantly improve when the fiber percentage is increased from 1.5 to 1.75 percent. When the fibers have a propensity to group to create yarn balls, this may be explained by raising the fiber content utilized to a certain value. As a result, the fiber distribution in concrete will be less even, which will lessen the impact of lowering fiber shrinkage [59]. In comparison to plain concrete specimens, all fiber-reinforced concrete samples with blended fibers showed considerably reduced shrinkage values up to 180 days. Fibers stop the development of microcracks on the concrete’s surface, which stops the movement of dangerous components in samples. As a consequence, the detrimental effects of shrinkage are minimized and the fracture density and size are decreased [61].

The research looked at the effects of steel and BF on the concrete’s dry shrinkage characteristics. Since the fibers will prevent the cementitious matrix from shrinking, shrinkage values should, in theory, decrease as fiber volume is raised. As the concrete has more water available to be utilized in the cement hydration process, enabling the concrete to not shrink for a longer length of time, the boost in the w/c percentage would result in a drop in shrinkage strain. With an increase in fiber content, shrinkage strain values reduced consistently across all fiber samples. Additionally, as compared to the same percentages of BFs, the usage of steel fibers revealed a much reduced shrinkage strain [62].

According to research fiber concrete had less shrinkage deformations than plain concrete without fibers, suggesting that it was more stable. By strengthening the bond among the fibers and mix, which facilitates the limiting of shrinkage during the drying activity, fibers may help reduce the amount of shrinkage in concrete [63].

Another study’s [64] investigation focused on the impact of GF on shrinkage. They found a significant reduction in initial shrinkage, which they ascribed to a solid link between the fibers and the cementitious mix. Additionally, the shrinkage of fiber-reinforced concrete was more uniform than that of fiber-free mortar. Finally, it was determined that fiber had a positive effect on containing fractures and lowering the likelihood that they would occur. Regarding confined shrinkage, it was noted in [65] that 1% fiber after 24 h decreased constrained stresses in mortar by 24%. It can be concluded that dry shrinkage cracks decreased considerably with the addition of BF due to the crack prevention of BF.

## 5. Electric Resistivity

The possibility of electrical charge transport through the composite is made clear by the material feature known as specific electrical resistivity. It often relies on the chemical makeup of the cementitious material, the kind and form of the pores, and the composition of the pore solution [66]. An essential factor characterizing a material’s capacity to conduct electric current is its electrical resistivity. Due to their insulating properties, dry cementitious materials display extremely high electrical resistivity values. Oven-dried concrete has an electrical resistance of roughly 109 Ω m [67].

**Figure 8:**
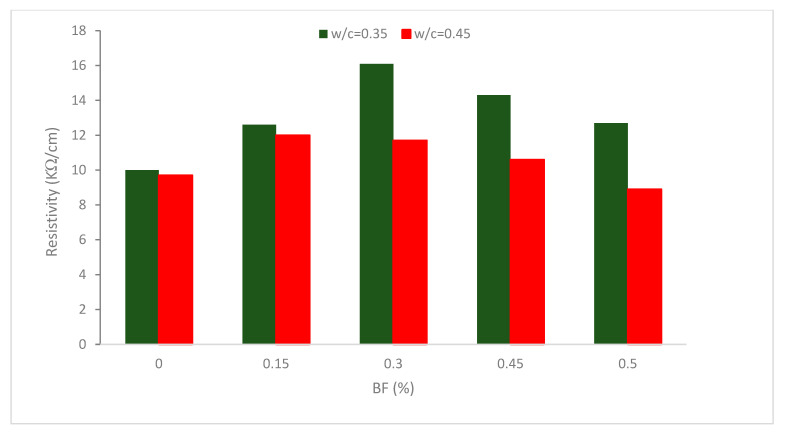
Resistivity of BF reinforced concrete: Source [62].

Figure 8 demonstrates that BF results in a decline in the penetrability of chloride ions (>12 kW/cm) for all combinations (w/c = 0.35) and a decline in the penetrability of chloride ions up to a 0.30% percent fiber for specimens with w/c = 0.45. Research also found that the presence of water and steel fiber clusters increased conductive pathways and lowered electrical resistance [67]. Due to the larger porosity gained due to a higher w/c ratio and fibers percentage, rising cavities through blending, and possible effects from the higher fiber volume, 0.45 and 0.50 percent. BF did not offer higher results for w/c = 0.45. Although there was no balling of the fibers, increased fiber volumes influence how the fibers are distributed, which reduces their effectiveness. According to research, BF increases water absorption and decreases electrical resistivity in cementitious materials, with the negative impact being more pronounced the greater the volume level of BF [41].

The samples with the biggest improvements were sample (w/c = 0.35) with 0.30 percent BFs and an increase in chloride ion penetration resistance of 61 percent, and sample (w/c = 0.45) with 0.45 percent BF and an increase in chloride ion penetration resistance of 47 percent. Due to the rise in the w/c ratio, sample (w/c = 0.35) results are higher than sample (w/c = 0.45) values, as predicted, and at 0.30 percent fiber offers the greatest improvement. Using steel fibers improved the resistance to chloride ion infiltration. This is because steel fibers interact with one another, increasing their susceptibility to assaults from chloride. The steel fiber reinforcement shows a 48.45 percent decline or a 0.50 percent decrease (w/c = 0.45) in chloride ion penetrability resistance.

According to research, adding fibers to concrete increased its electrical conductivity or decreased its electrical resistance. Particularly small fiber doses had a significant influence on the electrical resistivity that was measured, but larger fiber dosages made the changes less noticeable. The electrical resistance of the unreinforced concrete was split in half, from roughly 50 to 25 Ωm, with the addition of 30 kg/m^3^ of fiber. There is no linear relationship between fiber content and electrical resistivity; when an additional 30 kg/m^3^ of fibers were added, the loss of electrical resistivity was only around 30%. Contrarily, it was evident that conductivity almost linearly correlated with fiber concentration [68]. It can be concluded that BF increased electric resistivity. However, the increase depends on the BF percentages and w/c. Higher BF percentages or higher w/c results decrease in electric resistivity.

## 6. Freezing and Thawing

The compressive capacity is intimately connected with its porosity, the smaller the perviousness, the denser the composition, the greater the compressive capacity and the critical pore size and pore size distribution are directly correlated with the permeability and durability of concrete.

**Figure 9:**
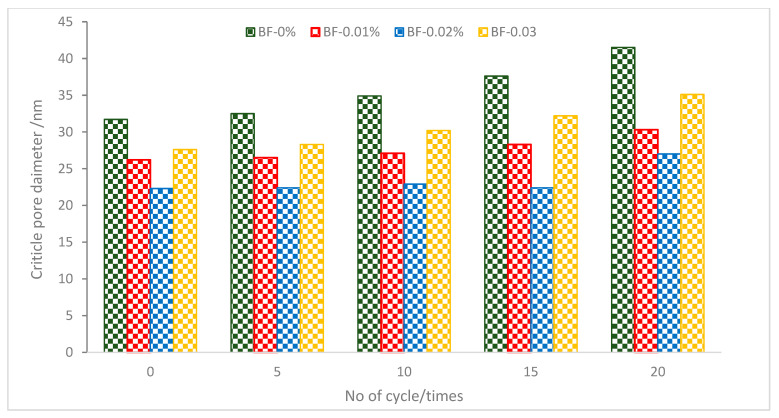
Porosity of BF reinforced concrete at different no. of cycle: Source [73].

Figure 9 implies that the initial porosity of the concrete will decrease with the addition of BF. It was discovered that the 0.02 percent BF group had the lowest original porosity, and the 0.03 percent BF group had a higher original porosity than the 0.02 percent BF and 0.02 percent BF groups. However, some studies claimed that promising results were achieved at 0.6% [69,70]. This was due to extreme fiber integration and significant amounts of disordered fiber clusters, which increased the likelihood of flaws. Each group’s concrete started to become more porous as the single-side salt–freezing–drying–wetting cycle developed.

The expansion tension brought on by sulphate crystallization and the tensile stress brought on by freezing pressure and capillary osmotic pressure enhanced the perviousness [71]. After 20 cycles, the perviousness with additions of 0, 0.01, 0.02, and 0.03 percent of BF rose by 9.8, 4.1, 4.7, and 7.5%, respectively. As can be observed, the inclusion of BF prevents the single-side salt–freezing–drying–wetting cycle from increasing perviousness. The addition of BF enhances the compactness and increases the depth and content of concrete’s resistance to sulphate ion destruction. BF resilience and crack resistance lessen the concentration of stress and the resulting damage. According to research, adding fiber to concrete may boost its resistance to freezing and thawing. Additionally, fibers may strengthen the matrix by halting the growth of cracks. Fibers may be added to boost the amount of unharmful openings, which can lower the extension pressure brought on by a freezing case and lower the amount of destruction from freezing–thawing [72].

The pore structure system in concrete is interconnected and dispersed at random. The critical pore diameter, which may indicate the connectedness of pores, is the maximum pore diameter that can link the bigger pores. The physical meaning is that pores cannot be linked to one another if the pore thickness is bigger than the critical aperture. The better the critical pore size, the more durable and impermeable the cement concrete pore arrangement.

**Figure 10:**
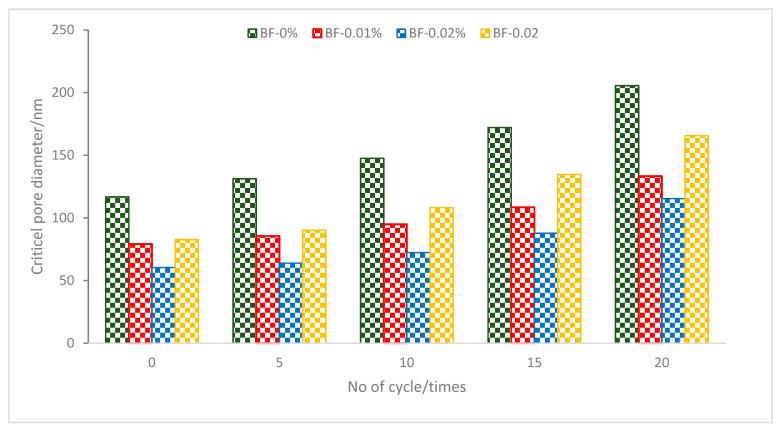
Critical pore diameter of BF reinforced concrete at different no. of cycle: Source [73].

Figure 10 displays the critical pore width for each group under various cycles. The critical pore width of concrete with additions of BF of 0, 0.01, 0.02, and 0.03 percent is 116.7, 79.1, 60.4, and 82.6 nm, respectively, under 0 cycles. The critical pore diameter of concrete is successfully decreased by the use of BF.

The critical pore size of the concrete rose progressively for each group as the cycle continued. The critical pore diameters for additions of 0, 0.01, 0.02, and 0.03 percent of BF were, respectively, 205.5, 133.3, 115.4, and 165.6 nm after 20 cycles. Concrete’s permeability resistance diminishes with an increase in critical pore size, making it less stable in service when subjected to a single cycle of salt, freezing, drying, and wetting. Each major cycle of control concrete saw a steady rise in growth rate. After 10 cycles, the growth rates of the concrete in the 0.01 and 0.02 percent BF groups greatly rose, but those in the 0.03 percent BF group notably increased after 5 cycles. It has been revealed that while BF has the impact of strengthening and crack resistance, the optimal performance still requires the synergistic action of SCM and the proper quantity of BF. The required pore width for concrete’s durability and impermeability is reliable with its macroscopic performance index in each cycle. It can be concluded that pore diameter decreased considerably with the addition of BF due to the crack prevention of BF. However, at a higher dose of BF (0.3%), the results increased in pore diameter value due to a lack of flowability, which increased compaction efforts, leading to more voids.

## 7. Chloride Content

**Figure 11:**
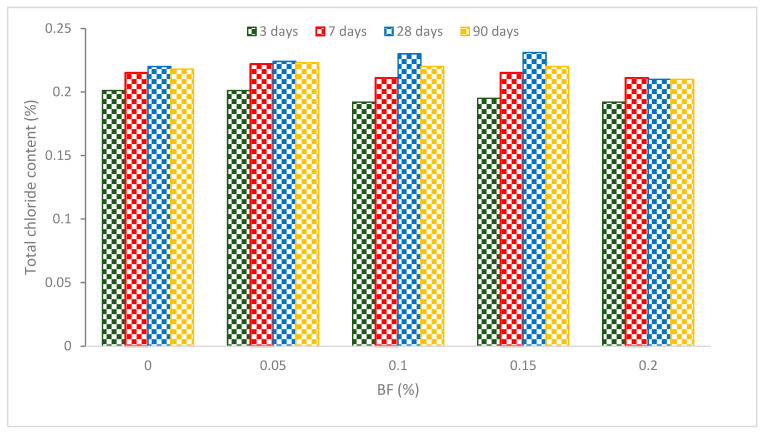
Total chloride content: Source [46].

The total chloride concentration of concrete after the addition of BF is shown in Figure 11. The concrete containing 0.05 percent fibers has a greater total chloride ion concentration than normal concrete after curing for more than 3 days. At 14 to 90 days, concrete reinforced with 0.2% BF had a lower total chloride ion level than other specimens. The total chloride ion level decreased by 0.48 to 5.8 percent after 3 days after adding 0.05 and 0.20 percent of BF. The amount of total chloride ions in concrete with 0.05 percent BF rose by 2.7 percent after 90 days. However, by adding 0.1 percent to 0.2 percent of BF, the total chloride ion level was reduced by 0.45 percent to 5 percent.

The pressure differential between the interior and exterior of the aggregate enables the prewetted water to be released when the moisture content of the concrete lowers due to continual hydration. Chloride ions from the inside of the aggregate with the water enter the concrete matrix as the internal curing process is accomplished. The internal curing action is most noticeable, the chloride ion concentration is maximum, and the pore structure is optimum in the 0.05 percent of BF-based concrete. A decreased total chloride ion concentration is the consequence of the internal curing action of concrete being less effective because of the greater BF content.

**Figure 12:**
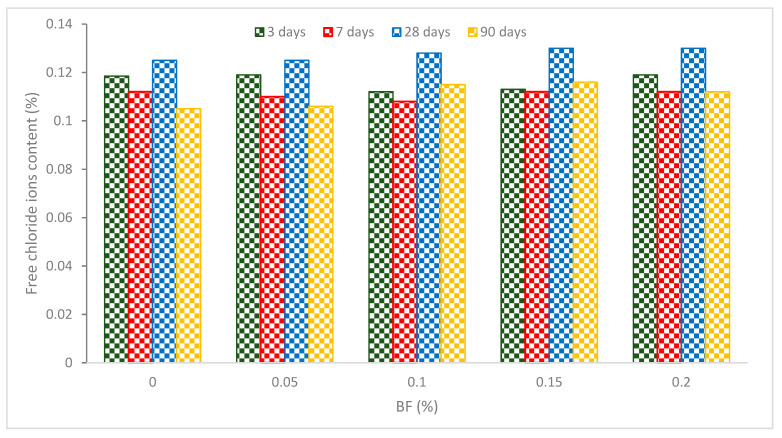
Free chloride content: Source [46].

The free chloride ion concentration of BF reinforced concrete reduces during the first stages of hydration as shown in Figure 12. However, the free chloride ion level rises around 14–28 days. All specimens had their greatest free chloride ion level at 28 days. All the BF-containing samples had greater free chloride ion contents than control concrete after 28 days, increasing by 1.6 percent to 4.8 percent. All specimens see a dramatic decline in free chloride ions after 60 days, which is 3.87 percent to 11.2 percent lower than at 28 days. The hydration of BF reinforced concrete has finished reducing after 90 days, and all specimens have stable free chloride ion contents of 0.12 percent to 0.14 percent, which is below the threshold chloride ion concentration for steel corrosion [74]. It can be concluded that the chloride ion content decreases with BF addition due to crack prevention, leading to more dense concrete. Furthermore, considerable improvement was observed particularly at later age curing (beyond 28 days).

## 8. Ultra-Sonic Pulse Velocity

**Figure 13:**
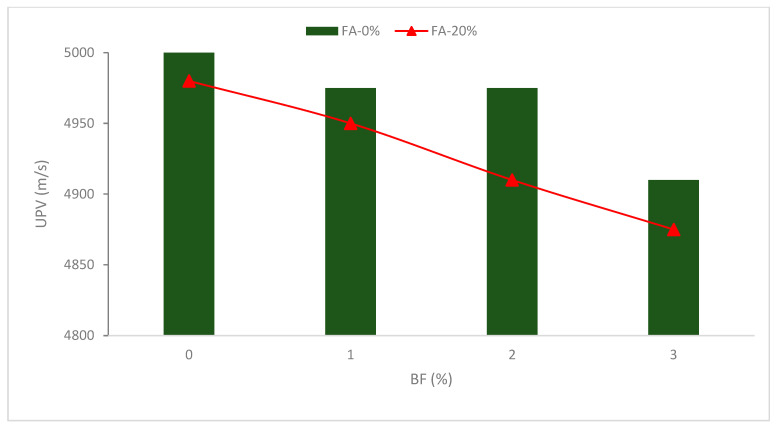
UPV of BF reinforced concrete: Source [75].

Figure 13 demonstrates that when the BF concentration increased, the pulse velocity decreased. The phenomenon may be attributed to several factors. The first is that BF-containing mixes have some capillaries that migrated into the hardened concrete as the hydration process progressed. It has long been known that the most significant influence on the impact of the transmission velocity of ultrasonic pulses comes from the capillaries in the concrete specimen. The fact that void concrete propagates pulses more quickly than solid matter does provide evidence that it slows the predicted velocity by increasing the pulse’s capillary transit [75].

The network structure created by BF additionally strengthens the matrix’s internal binding force and prevents cement paste from flowing or segregating. As a result, the existence of BF reduces the flowability of concrete [41], making it difficult to fill gaps in concrete with vibration. As a consequence, the compactness reduces and the interior porosity rises, which lowers the VPV. Furthermore, with the same fiber content, the UPV of a combination including cementitious materials is always smaller than that of a blend without them. This is explained by the fact that cement has greater specific gravity than fly ash. The compaction process becomes more challenging at higher dosages, such as 4.0 percent fiber replacement, leading to porous concrete and a lower UPV. Reinforced concrete has less homogeneity than regular concrete. The UPV declined as the quantity of fiber improved. Reinforced SCC showed superior homogeneity, while steel fiber had a lesser pulse velocity than concrete with GF [76]. It can be concluded that BF decreased the UPV value due to internal porosity caused by a lack of flowability.

## 9. Alkali Resistance

The challenge of excessive alkalinity from the adjacent material is a major problem when the fiber reinforced concrete components are buried in the concrete using the near-surface mounting technique. As concrete is alkaline, the alkali resistance of BF used in concrete is crucial. The research findings on BF’s alkali resistance are contradictory. According to research, BF has strong alkali resistance at both low and high temperatures [77]. Similar research established that BF’s alkali resistance is superior to its acid resistance [78].

**Figure 14:**
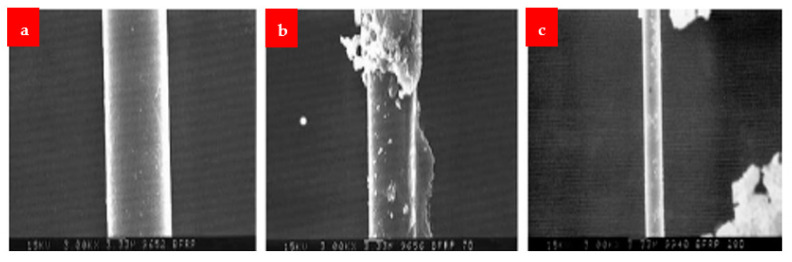
BF (**a**) normal, (**b**) 7 days under NaOH and (**c**) 28 days under NaOH [30].

**Figure 15:**
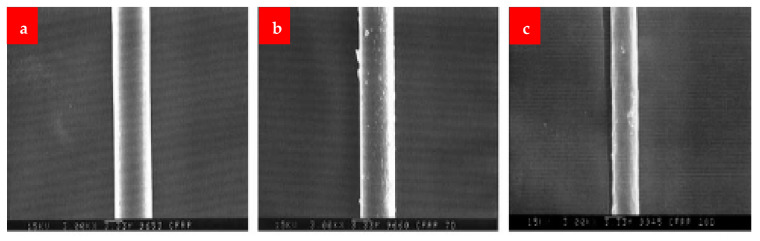
CF (**a**) normal, (**b**) 7 days under NaOH and (**c**) 28 days under NaOH [30].

**Figure 16:**
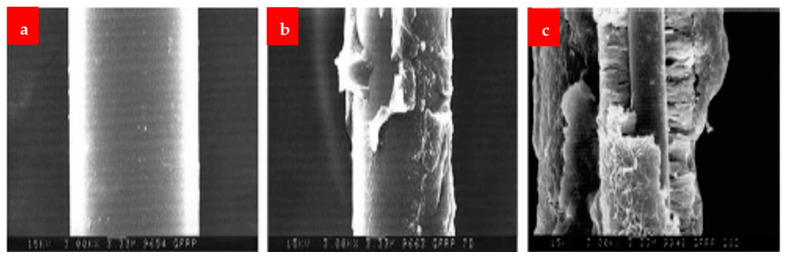
GF (**a**) normal, (**b**) 7 days under NaOH and (**c**) 28 days under NaOH [30].

The SEM images of the fibers after dipping at different mediums are shown in Figure 14, Figure 15 and Figure 16. Under alkaline conditions, it was found that both BFs and GFs dramatically dropped volume. As the immersion duration lengthened, reaction products formed on the surfaces of these two fibers and disintegrated, reducing the sound portion or volume of the fibers. These reaction products were thought to be the result of the reaction among the alkali solution and the fibers’ SiO_2_ content. No such reaction product was seen in the CF, and the projected volume decrease after 28 days of immersion was less than 20%. The strength fluctuated in relation to the duration of immersion. Over 28 days, the capacity of the glass and BF decreased by more than 80%. After the CF had been submerged for 28 days, a strength drop of roughly 13 percent was recorded.

According to the research [34], BF is more resistant to corrosion in saltwater than CF and GF. Contrary to these observations, some researchers noted that BF’s alkali resistance was low. According to Lee’s research, up to 40% of the mass may be lost in strong alkaline solutions. The strength loss rate of BF after 90 days of being saturated in an alkaline mixture might reach 80% [35].

**Figure 17:**
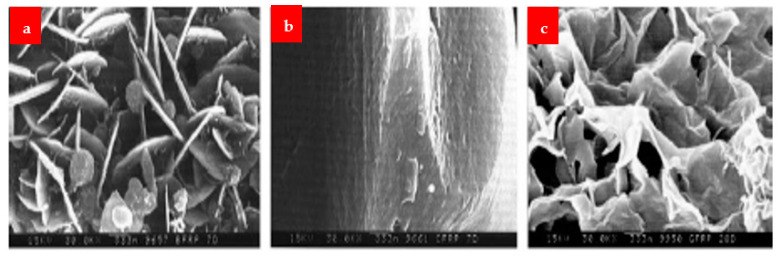
Alkali reaction product of (**a**) BF, (**b**) CF, and (**c**) GF [30].

Figure 17 displays SEM pictures of the reaction products that formed on the fibers’ surface at a temperature of 30 K. The effects were not found in the CF, but the basalt and the GFs exhibited plate-shaped structures. This structure seems to be susceptible to the solution flow, allowing for the continual growth and accumulation of products on the surface. The basalt and GFs seem to have comparable deteriorating attributes and lose capacity and volumetric constancy more quickly than the CF in a critical alkali situation, according to this alkali-resistance experiment.

**Figure 18:**
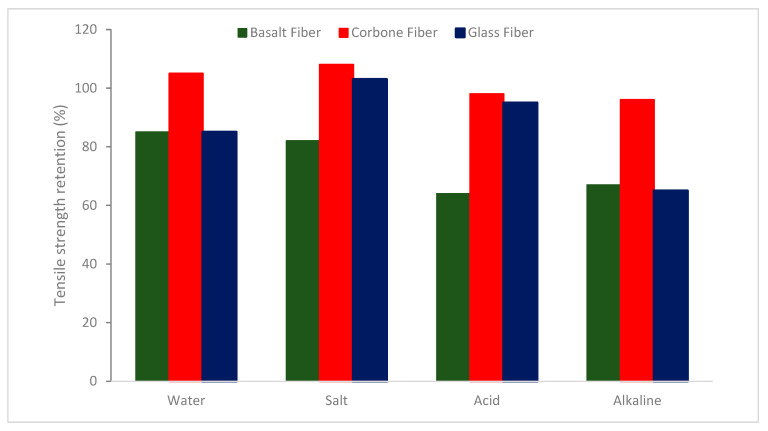
Tensile strength retention of different fibers after degradation: Source [34].

Assessments of the tensile capacity of various fibers after 66 days of exposure are displayed in Figure 18. Figure 18 demonstrates that whereas the tensile capacity of the BFs subjected to the acid and alkaline solutions was virtually totally reduced, the tensile capacity of the CFs subjected to all kinds of solutions remained unchanged. The GFs had substantially greater acid resistance than the BFs, although they degraded much more quickly in water and salt solutions. It can be concluded that BF shows comparable resistance to water, salt, and alkaline corrosion, but weaker resilience to acid corrosion than GFs and CFs.

**Figure 19:**
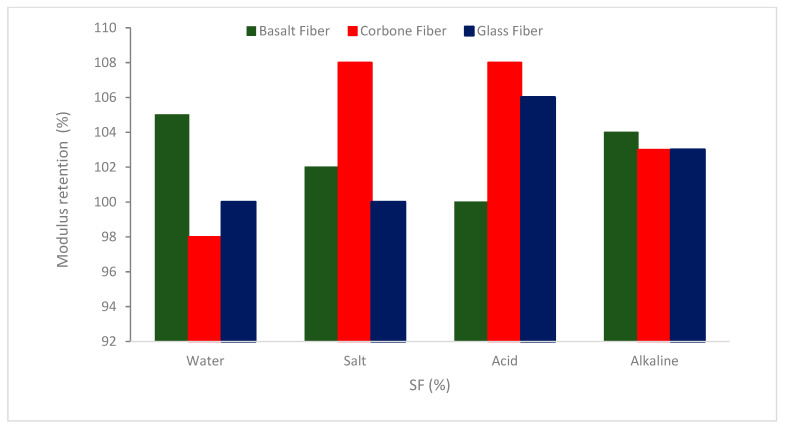
Elastic modulus retention of different fibers after degradation: Source [34].

Figure 19 shows how basalt, carbon, and GFs subjected to water and salt solutions retain their high modulus properties similarly. While the modulus of the CFs exposed to the acid and alkaline mixtures was almost unaffected, the modulus of the basalt and GFs degraded in a manner comparable to how their tensile capacity degraded after being subjected to the solutions.

## 10. Conclusions

Basalt fibers (BFs) may help to enhance the pore size distribution of concrete. Concrete durability properties are therefore significantly increased by BF, which may minimize fractures and stop water or hazardous ions from infiltrating concrete. In this analysis, the effects of BFs on the durability assets of concrete are summarized. The detailed conclusions are given below.

- The apparent density and ultra-sonic pulse velocity exhibit a decreasing trend with increasing BF volume content.
- Shrinkage declined with the addition of BF. It is owing to the crack’s prevention and higher elastic modulus of BF.
- Surface resistivity boosted with BF up to 0.3%. However, beyond the accumulation of BF, the results declined in surface resistivity due to a lack of flowability.
- The rapid chloride ions penetrability of concrete increased with the addition of BFs due to a weak fiber-matrix interfacial transition zone (fiber-ITZ) with extreme permeability. However, the addition of mineral admixture decreased the rapid chloride ions penetration up to some extent due to pozzolanic and micro fill-up cavities. Though data are fewer, and additional details analyses are necessary.
- The freezing and thawing resistance of concrete was boosted with the addition of BF. The highest resistance was detected at a 0.02% addition of BF. However, further addition of BF resulted in a decrease in freezing and thawing resistance due to a lack of flowability.
- BFs show comparable resistance to water, salt, and alkaline corrosion, but weaker resilience to acid corrosion than GFs and CFs. However, the statement is not clear, as some studies conclude that the BF is more corrosion resistant than carbon and GFs.

Overall, less knowledge is accessible on the durability characteristics of BFs-reinforced concrete. Furthermore, some studies claim improvements in durability assets of concrete reinforced with BF, while other findings claimed decreased durability properties of concrete reinforced with BF. Thus, the review suggests a detailed study on the durability aspects of BFs-reinforced concrete. Additionally, no information is available on the creep properties of BFs-reinforced concrete. Finally, the review also suggests a detailed study on the corrosion resistance of BF by adjusting the matrix and the coupling agent to enhance the corrosion endurance of BF-reinforced concrete.

## Data Availability

All the data available in main text.
